# Genetic dissection of the fatty liver QTL *Fl1sa* by using congenic mice and identification of candidate genes in the liver and epididymal fat

**DOI:** 10.1186/s12863-016-0453-7

**Published:** 2016-11-17

**Authors:** Miyako Suzuki, Misato Kobayashi, Tamio Ohno, Shinsaku Kanamori, Soushi Tateishi, Atsushi Murai, Fumihiko Horio

**Affiliations:** 1 Department of Applied Molecular Biosciences, Graduate School of Bioagricultural Sciences, Nagoya University, Nagoya, 464-8601 Japan; 2 Division of Experimental Animals, Center for Promotion of Medical Research and Education, Graduate School of Medicine, Nagoya University, Nagoya, 466-8550 Japan

**Keywords:** Fatty liver, Congenic, *fl1sa*, Epididymal fat, Interaction, QTL, Candidate gene, Mouse

## Abstract

**Background:**

Nonalcoholic fatty liver disease (NAFLD) is a multifactorial disease caused by interactions between environmental and genetic factors. The SMXA-5 mouse is a high-fat diet-induced fatty liver model established from SM/J and A/J strains. We have previously identified *Fl1sa*, a quantitative trait locus (QTL) for fatty liver on chromosome 12 (centromere-53.06 Mb) of SMXA-5 mice. However, the chromosomal region containing *Fl1sa* was too broad. The aim of this study was to narrow the *Fl1sa* region by genetic dissection using novel congenic mice and to identify candidate genes within the narrowed *Fl1sa* region.

**Results:**

We established two congenic strains, R2 and R3, from parental A/J-12^SM^ and A/J strains. R2 and R3 strains have genomic intervals of centromere-29.20 Mb and 29.20–46.75 Mb of chromosome 12 derived from SM/J, respectively. Liver triglyceride content in R2 and R3 mice was significantly lower than that in A/J mice fed with a high-fat diet for 7 weeks. This result suggests that at least one of the genes responsible for fatty liver exists within the two chromosomal regions centromere-29.20 Mb (R2) and 29.20–46.75 Mb (R3). We found that liver triglyceride accumulation is inversely correlated with epididymal fat weight among the parental and congenic strains. Therefore, the ectopic fat accumulation in the liver may be due to organ-organ interactions between the liver and epididymal fat. To identify candidate genes in *Fl1sa*, we performed a DNA microarray analysis using the liver and epididymal fat in A/J and A/J-12^SM^ mice fed with a high-fat diet for 7 weeks. In epididymal fat, mRNA levels of *Zfp125* (in R2) and *Nrcam* (in R3) were significantly different in A/J-12^SM^ mice from those in A/J mice. In the liver, mRNA levels of *Iah1* (in R2) and *Rrm2* (in R2) were significantly different in A/J-12^SM^ mice from those in A/J mice.

**Conclusions:**

In this study, using congenic mice analysis, we narrowed the chromosomal region containing *Fl1sa* to two regions of mouse chromosome 12. We then identified 4 candidate genes in *Fl1sa*: *Iah1* and *Rrm2* from the liver and *Zfp125* and *Nrcam* from epididymal fat.

**Electronic supplementary material:**

The online version of this article (doi:10.1186/s12863-016-0453-7) contains supplementary material, which is available to authorized users.

## Background

Nonalcoholic fatty liver disease (NAFLD) is a multifactorial disease caused by interactions between environmental and genetic factors. NAFLD is frequently complicated by type 2 diabetes, obesity, insulin resistance, and hyperlipidemia. In developed countries, the prevalence of NAFLD reached approximately 30% of adults [1, 2]. Environmental factors such as high-fat diets, methionine/choline-deficient diets, and low-carbohydrate (ketogenic) diets can induce development of NAFLD [3, 4]. In humans, *APOC3* variants, *PLIN1* mutations, and *PNPLA3* variants have been reported as genetic factors of NAFLD [1, 5]. However, the underlying mechanism of how environmental and genetic factors interact to cause NAFLD is largely unknown.

SMXA-5 mouse is one of the SMXA recombinant inbred (RI) strains established from parental SM/J and A/J strains [6]. We have previously found that SMXA-5 mice developed severe fatty liver on a high-fat diet, although parental SM/J and A/J mice were resistant to fatty liver [7]. To elucidate the genetic mechanism of NAFLD in SMXA-5 mice, we performed a quantitative trait locus (QTL) analysis using (SM/J × SMXA-5)F2 intercrossed mice and identified a significant QTL (*Fl1sa*, ***f*** *atty* ***l*** *iver* ***1*** *in* ***S*** *MX* ***A*** *RI strains*) for liver weight, liver triglyceride, and liver total lipid content on centromere-53.06 Mb of mouse chromosome 12 [8]. This QTL for the development of fatty liver was attributed to the A/J allele. To confirm the effect of *Fl1sa*, we analyzed the fatty liver phenotypes in A/J-12^SM^ chromosomal substitution (consomic) mice, in which chromosome 12 of the SM/J mouse was introduced into the A/J mouse genome. Consequently, we demonstrated that liver total lipid, liver triglyceride, and liver weight in A/J-12^SM^ mice were significantly lower than those in A/J mice on a high-fat diet for 7 weeks, but not lower than those in mice on the normal diet [9]. We verified the effect of the A/J-derived *Fl1sa* on the development of the high-fat diet-induced fatty liver. We also identified three candidate genes, *Iah1*, *Rrm2*, *Prkd1*, in *Fl1sa* by DNA microarray analysis using livers of A/J and A/J-12^SM^ mice fed on a high-fat diet.

**Fig. 1:**
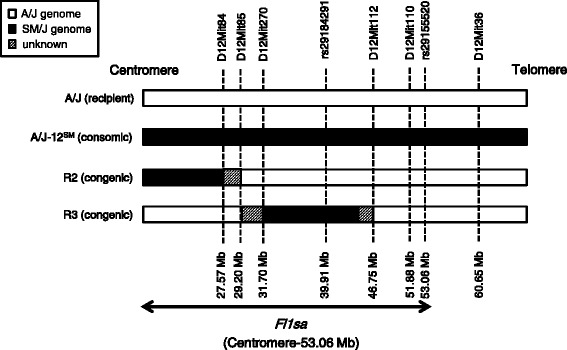
Chromosome 12 constructs of consomic and congenic strains. The genomes of consomic and congenic strains consist of recipient A/J and donor SM/J genomes. White and black boxes represent A/J and SM/J genomic intervals, respectively. Gray boxes represent unclear regions where the genomic intervals were derived from the A/J or SM/J strains. Arrow represents the *Fl1sa* region (centromere-53.06 Mb) on chromosome 12

In this study, to narrow the chromosomal region of *Fl1sa*, first, we constructed two strains of congenic mice, R2 and R3, from parental A/J and A/J-12^SM^ strains (Fig. 1), and then analyzed the lipid accumulation in their liver. As the liver triglyceride content in R2 and R3 congenic mice was significantly lower than that in A/J mice, the *Fl1sa* region was narrowed to two parts of chromosome 12. Subsequently, in both the narrowed *Fl1sa* regions, we attempted to identify candidate genes in *Fl1sa* using DNA microarray analyses of liver and epididymal fat from A/J and A/J-12^SM^ mice. As a result, we identified several candidate genes in both the liver and epididymal fat.

## Methods

### Animals

The A/J strain was purchased from Japan SLC (Hamamatsu, Japan) and maintained in our facility at the Graduate School of Bioagricultural Sciences, Nagoya University. The A/J-12^SM^ strain (chromosome 12 consomic mouse) was constructed from the A/J (recipient) and SM/J strain (donor) at the Institute for Laboratory Animal Research (Nagoya University) as previously described [10]. The R2 and R3 congenic strains were constructed from A/J (recipient) and A/J-12^SM^ strains (Fig. 1). Practically, to produce N1 mice that were heterozygous for the SM/J-derived chromosome 12 on the background strain A/J, male A/J-12^SM^ mice were mated with female A/J mice. The N1 mice were then backcrossed to A/J mice to produce heterozygous mice carrying novel genomic intervals of interest on chromosome 12. Heterozygous mice were mated with A/J mice and their progeny with the same genomic intervals were obtained. Finally, to generate mice carrying homozygous novel genomic intervals, the heterozygous mice were intercrossed. Mouse tails were collected under anesthesia induced using isoflurane. Genomic DNA was extracted from tails using the DNeasy Blood & Tissue kit (QIAGEN). Microsatellite markers and single nucleotide-polymorphisms (SNPs) were used for genotyping of R2 and R3 genomic DNA (Additional file 1). The physical positions of microsatellite markers and SNPs were taken from the Ensembl database (GRCm38.P4). We previously mapped the QTL (*Fl1sa*) for fatty liver by using male (SM/JxSMXA-5)F2 mice [8]. Subsequently, we have confirmed that the *Fl1sa* contributed to fatty liver traits in male A/J-12^SM^ consomic mice [9]. Therefore, in this study, all procedures were performed by using only male mice. All mice were maintained in our facilities at a temperature of 23 ± 2 °C, humidity of 55 ± 5%, and a light/dark cycle of 12 h. Mice were weaned at 3 weeks of age and housed at one animal per cage. All mice had access to food and tap water *ad libitum*.

### Experimental schedule and diet composition

Male A/J, A/J-12^SM^, R2, and R3 mice were fed CE-2 standard chow (CLEA Japan, Inc., Japan) until 6 weeks of age, thereafter fed a high-fat diet (D07053003; Research Diets, New Brunswick, NJ, USA) from 6 to 13 weeks of age. The composition (per kg diet) of the high-fat diet was as follows: casein, 209 g; carbohydrate (corn starch: sucrose: maltodextrin 10, 94:100:175), 369 g; mineral mix S10022G, 35 g; vitamin mix V10037, 10 g; choline bitartrate, 2 g; corn oil, 35 g; lard, 300 g; and cellulose BW200, 40 g. The content of fat in this diet was 56% (energy %). The body weight and food intake were measured every week during the experimental period (6–13 weeks of age). At 13 weeks of age, all mice were sacrificed by cervical dislocation after 4-h fast (9:00–13:00 h). The liver and white adipose tissue (subcutaneous fat, epididymal fat, mesenteric fat, and retroperitoneal fat) were harvested, weighed, and immediately frozen using liquid nitrogen. Blood samples were collected from orbital veins to measure serum lipids. All procedures and animal care were approved by the Animal Experiment Committee, Graduate School of Bioagricultural Sciences, Nagoya University (approval No. 2013021803, 2014020403, 2015022603) and were carried out in accordance with the Regulations on Animal Experiments of Nagoya University.

### Measurement of serum triglyceride and cholesterol

Serum triglyceride and cholesterol concentrations were measured using the Triglyceride-E test Kit (Wako Pure Chemical Industries, Japan) and the Cholesterol-E test Kit (Wako Pure Chemical Industries, Japan), respectively.

### Hepatic triglyceride and total lipid content analysis

Frozen livers (approximately 0.5 g each) were homogenized using chloroform-methanol (2:1), and statically extracted overnight. A portion of the organic extract was dried, and the hepatic triglyceride content was measured using the Triglyceride-E test Kit. The remaining organic solvent was used for total liver lipid measurement as previously described by Folch et al. [11].

### DNA microarray analysis in epididymal fat

Total RNA was isolated using the TRI reagent (Molecular Research Center Inc.) and RNeasy Mini Kit (QIAGEN) from frozen epididymal fat obtained from 4-h fasted A/J and A/J-12^SM^ male mice that were fed the high-fat diet for 7 weeks. Total RNA from three mice per strain was pooled for each chip. Whole transcripts from epididymal fats were measured using a Mouse Genome ST 2.0 array (Affymetrix). Raw data were normalized with RMA-sketch algorithm by Affymetrix Expression Console Software ver.1.3.0. The microarray data have been deposited in the NCBI Gene Expression Omnibus (GEO) (GSE79281). The details of expression profiles are shown in Additional file 2.

### Real-time RT-PCR

Total RNA was isolated using the TRI reagent from frozen liver and epididymal fat of A/J, A/J-12^SM^, R2, and R3 male mice that were fed the high-fat diet for 7 weeks. Isolated RNA was then treated with TURBO DNA-free kit (Ambion) to eliminate DNA contamination. Thereafter, the cDNA was synthesized from DNase-treated total RNA using the High Capacity Reverse Transcription kit (Applied Biosystems). Gene expression was determined using the StepOnePlus^TM^ Real-Time PCR System (Applied Biosystems) with the Thunderbird qPCR Mix or the Thunderbird SYBR qPCR Mix (TOYOBO, Japan). Each mRNA level was normalized to the corresponding *β-actin* mRNA level. To determine the mRNA level of *Iah1*, we used TaqMan probes (TaqMan Gene Expression Assays, Mm00509467_m1; Applied Biosystems). The details of primers used for the SYBR Green assays are shown in Additional file 3.

### Statistical analysis

All results were expressed as mean ± SEM. One-way ANOVA and subsequent Dunnett’s test were used to compare the means of A/J-12^SM^, R2, and R3 with those of A/J mice. Student’s *t*-test was used to compare the means between A/J and A/J-12^SM^ mice. The correlation between fatty liver parameters (liver weight and liver triglyceride content) and epididymal fat weight were analyzed using Spearman correlation analysis. Differences with *p* < 0.05 were regarded as significant. Statistical analyses were performed by using StatView 5.0 software (SAS Institute, Cary, NC).

## Results

### Phenotypic analysis in A/J, A/J-12^SM^, R2, and R3 mice that were fed the high-fat diet for 7 weeks

**Fig. 2:**
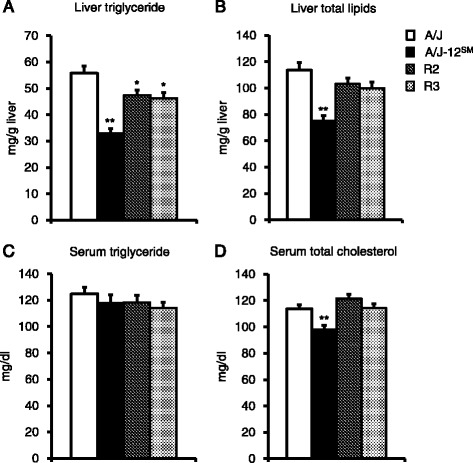
Liver lipids and serum lipids of A/J, A/J-12^SM^, and congenic strains. **a** Liver triglyceride concentration, **b** Liver total lipid concentration, **c **Serum triglyceride concentration, and **d** Serum total ﻿cholesterol concentraion of A/J, A/J-12^SM^, and congenic strains fed the high-fat diet for 7 weeks (*n* = 14–16, *p < 0.05, **p < 0.01 versus A/J mice by Dunnett’s test)

Although initial body weight and food intake were not different in each strain, the final body weight in A/J-12^SM^ and R3 mice was significantly lower than that in A/J mice (Table 1). Liver and mesenteric fat weights in A/J-12^SM^ mice were significantly lower than those in A/J mice. On the other hand, epididymal fat and retroperitoneal fat weight in A/J-12^SM^ mice were significantly higher than those in A/J mice. R2 and R3 mice did not show any significant differences in tissue weight compared to A/J mice. However, liver weight in R2 and R3 mice tended to be slightly lower than that in A/J mice. In addition, epididymal fat weight in R2 mice tended to be higher than that in A/J mice. Liver triglyceride content was significantly lower in A/J-12^SM^, R2, and R3 mice, compared with that in A/J mice (Fig. 2a). The changes in liver total lipid content in all strains were similar to those in liver triglyceride content (Fig. 2b). Liver triglyceride and liver total lipid in R2 and R3 mice showed intermediate values between those of A/J and A/J-12^SM^ mice. These results suggest that the genes responsible for fatty liver exist in the centromere-29.20 Mb (SM/J region in R2 strain) and 29.20–46.75 Mb (SM/J region in R3 strain) regions of chromosome 12, respectively (Fig. 1). Serum triglyceride concentration did not differ among all strains. Serum total cholesterol concentration in A/J-12^SM^ mice was significantly lower than that in A/J mice; however, there were no differences in R2 and R3 mice relative to the A/J mice (Fig. 2c and d).

**Table 1:** Body weight, food intake, and tissue weight in A/J, A/J-12^SM^ consomic mice and R2 and R3 congenic mice fed the high-fat diet

	A/J	A/J-12^SM^	R2	R3
Body weight (g)				
0 weeks of feeding with the HFD (Initial)	21.9 ± 0.3	22.1 ± 0.3	21.4 ± 0.4	21.2 ± 0.5
7 weeks of feeding with the HFD (Final)	39.4 ± 0.4	35.8 ± 0.7**	38.2 ± 0.9	35.6 ± 0.7**
Food intake (g/g BW/day)^a^				
3 weeks of feeding with the HFD	0.065 ± 0.003	0.065 ± 0.001	0.065 ± 0.002	0.071 ± 0.003
6 weeks of feeding with the HFD	0.053 ± 0.002	0.058 ± 0.001	0.052 ± 0.001	0.056 ± 0.002
Weight of tissues (g/100 g BW)				
Liver	3.66 ± 0.07	2.99 ± 0.08**	3.40 ± 0.07	3.47 ± 0.10
Subcutaneous fat^b^	3.86 ± 0.12	3.48 ± 0.12	3.73 ± 0.19	3.55 ± 0.17
Epididymal fat	4.82 ± 0.18	6.24 ± 0.21**	5.35 ± 0.16	4.97 ± 0.18
Mesenteric fat	3.60 ± 0.13	2.90 ± 0.11**	3.56 ± 0.17	3.32 ± 0.11
Retroperitoneal fat	1.26 ± 0.06	1.52 ± 0.04**	1.20 ± 0.04	1.40 ± 0.07

Each value is expressed as the mean ± SEM

*n* = 13–16, ** *p* < 0.01, significant difference versus A/J strain by Dunnett’s test

^a^BW, body weight

^b^Subcutaneous fat was dissected between the root of the forefoot and the hind leg on right side of the body

### DNA microarray analysis of epididymal fat in A/J mice and A/J-12^SM^ mice

**Fig. 3:**
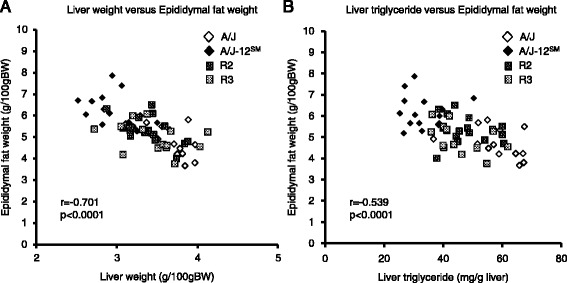
Correlation of fatty liver phenotype with epididymal fat weight. **a** A scatter plot of liver weight versus epididymal fat weight and **b** a scatter plot of liver triglyceride versus epididymal fat weight. Data were pooled from all mice fed the high-fat diet for 7 weeks (*n* = 59, correlation coefficient and *p*-value were calculated by Spearman correlation analysis)

**Fig. 4:**
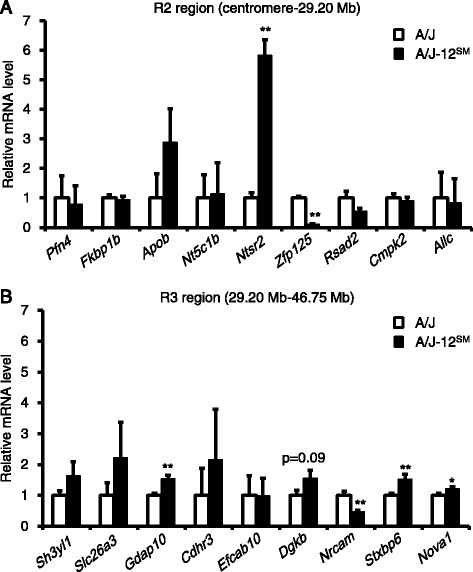
mRNA levels of differentially expressed genes existing in the genomic region of R2 or R3 congenic strains. Genes differentially expressed in epididymal fat between A/J and congenic mice were selected from a DNA microarray analysis (Table 2). The mRNA levels of selected genes exist in the genomic region of R2 congenic (**a**) or R3 congenic (**b**) strains. The mRNA levels of selected genes were measured using real-time RT-PCR. Epididymal fats were collected from A/J or A/J-12^SM^ mice fed the high-fat diet for 7 weeks (*n* = 5–6, *p < 0.05, **p < 0.01 versus A/J strain by Student’s *t*-test)

Liver triglyceride and total lipid in A/J-12^SM^ mice were significantly lower than those in A/J mice (Fig. 2a and b). An inverse correlation in tissue weight was observed between epididymal fat and the liver (*r* = −0.701, *p* < 0.0001, Fig. 3a). In addition, there was an inverse correlation between epididymal fat weight and liver triglyceride (*r* = −0.539, *p* < 0.0001, Fig. 3b). These results suggest that epididymal fat is an important tissue for the development of fatty liver. We then performed DNA microarray analysis in A/J and A/J-12^SM^ mice using total RNA obtained from epididymal fat. We identified genes whose expression levels were changed <0.60-fold or >1.68-fold in A/J-12^SM^ mice compared to those in A/J mice (Additional file 2 and Table 2). In epididymal fat, 19 genes were differentially expressed between the A/J and A/J-12^SM^ mice in the centromere-46.75 Mb region on chromosome 12. The genes *Pfn4*, *Fkbp1b*, *Apob*, *Nt5c1b*, *Ntsr2*, *Zfp125*, *Rsad2*, *Cmpk2,* and *Allc* were found in the R2 interval (centromere-29.20 Mb) on chromosome 12 (Table 2). Furthermore, the genes *Sh3yl1*, *Slc26a3*, *Gdap10*, *Cdhr3*, *Efcab10*, *Mir680-3*, *Dgkb*, *Nrcam*, *Stxbp6,* and *Nova1* were found in the R3 interval (29.20–46.75 Mb) on chromosome 12. In order to validate the expression of these genes, we performed real-time RT-PCR (except for Mir680-3, because it is a micro-RNA). We confirmed significant differences in gene expression levels of 6 genes (*Ntsr2*, *Zfp125*, *Gdap10*, *Nrcam*, *Stxbp6,* and *Nova1*) between A/J and A/J-12^SM^ mice (Fig. 4a and b). Thus, we identified 6 candidate genes from epididymal fat.

**Table 2:** Up-regulated and down-regulated genes in centromere-46.75 Mb (R2 and R3 regions) of chromosome 12 in the epididymal fat in the A/J-12^SM^ consomic strain

Symbol	Gene name	Position (Mbp)	Region	Fold change^a^	Gene bank ID
*Pfn4*	profilin family, member 4	4.76	R2	0.525	NM_028376
*Fkbp1b*	FK506 binding protein 1b	4.83	R2	0.557	NM_016863
*Apob*	apolipoprotein B	7.97	R2	7.460	NM_009693
*Nt5c1b*	5’-nucleotidase, cytosolic IB	10.36	R2	0.296	NM_027588
*Ntsr2*	neurotensin receptor 2	16.65	R2	1.896	NM_008747
*Zfp125*	zinc finger protein 125	20.89	R2	0.104	AJ005350
*Rsad2*	radical S-adenosyl methionine domain containing 2	26.44	R2	0.580	NM_021384
*Cmpk2*	cytidine monophosphate (UMP-CMP) kinase 2, mitochondrial	26.46	R2	0.463	NM_020557
*Allc*	allantoicase	28.55	R2	0.366	NM_053156
*Sh3yl1*	Sh3 domain YSC-like 1	30.91	R3	0.565	NM_013709
*Slc26a3*	solute carrier family 26, member 3	31.39	R3	0.361	ENSMUST 00000001254
*Gdap10*	ganglioside-induced differentiation-associated-protein 10	32.82	R3	1.928	BC052902
*Cdhr3*	cadherin-related family member 3	33.03	R3	0.557	NM_001024478
*Efcab10*	EF-hand calcium binding domain 10	33.39	R3	0.578	NM_029152
*Mir680-3*	microRNA 680-3	35.19	R3	2.545	NR_030449
*Dgkb*	diacylglycerol kinase, beta	37.88	R3	1.879	NM_178681
*Nrcam*	neuronal cell adhesion molecule	44.32	R3	0.529	NM_176930
*Stxbp6*	syntaxin binding protein 6 (amisyn)	44.85	R3	3.307	NM_144552
*Nova1*	neuro-oncological ventral antigen 1	46.69	R3	1.908	NM_021361

Up-regulated (log2^0.75^, >1.68-fold) and down-regulated (log2^-0.75^, <0.60-fold) genes were identified from a DNA microarray analysis between A/J and A/J-12^SM^ mice

^a^Fold change was calculated by the gene expression level in A/J-12^SM^ relative to that in A/J mice

### Expression levels of candidate genes in congenic strains

**Fig. 5:**
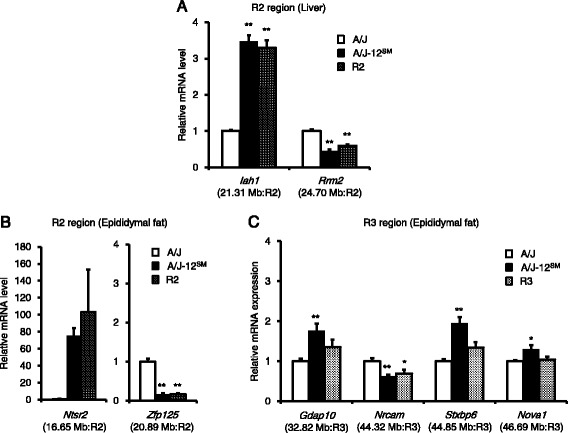
mRNA levels of candidate genes in two congenic strains. The mRNA levels of candidate genes in liver (**a**) and epididymal fat (**b** and **c**) were measured using real-time RT-PCR. A/J, A/J-12^SM^, R2, or R3 mice were fed the high-fat diet for 7 weeks (*n* = 6–8, *p < 0.05, **p < 0.01 versus A/J mice by Dunnett’s test). Physical positions and the congenic regions of each gene are given in parentheses

We previously performed a DNA microarray analysis using mRNA from the liver and succeeded in identifying candidate genes *Iah1* and *Rrm2* in the R2 region (data have been deposited in the NCBI GEO (GSE67340)) [9]. Furthermore, in this study, we succeeded in identifying candidate genes *Ntsr2*, *Zfp125*, *Gdap10*, *Nrcam*, *Stxbp6,* and *Nova1*, in epididymal fat from A/J and A/J-12^SM^ mice by DNA microarray analysis and real-time RT-PCR (Fig. 4). Subsequently, we analyzed the mRNA levels of candidate genes in congenic mice. In the liver, *Iah1* (isoamyl acetate-hydrolyzing esterase 1 homolog *(Saccharomyces cerevisiae)*; 21.31 Mb in the R2 region) mRNA level was significantly higher in A/J-12^SM^ and R2 mice than in A/J mice (Fig. 5a). *Rrm2* (Ribonucleotide reductase M2; 24.70 Mb in R2 region) mRNA level was significantly lower in A/J-12^SM^ and R2 mice than in A/J mice. In the epididymal fat, *Ntsr2* (neurotensin receptor 2; 16.65 Mb in R2 region) mRNA levels tended to be higher in A/J-12^SM^ and R2 mice than in A/J mice (Fig. 5b). *Zfp125* (Zinc finger protein 125; 20.89 Mb in R2 region) mRNA levels were significantly lower in A/J-12^SM^ and R2 mice than in A/J mice. *Gdap10* (ganglioside-induced differentiation-associated-protein 10; 32.82 Mb in R3 region), *Stxbp6* (syntaxin binding protein 6 (amisyn); 44.85 Mb in R3 region), and *Nova1* (neuro-oncological ventral antigen 1; 46.69 Mb in R3 region) mRNA levels were significantly different in A/J-12^SM^ mice, but not in R3 mice compared to those in A/J mice (Fig. 5c). *Nrcam* (neuronal cell adhesion molecule; 44.32 Mb in R3 region) mRNA levels were significantly lower in A/J-12^SM^ and R3 mice than in A/J mice. In summary, only *Zfp125* mRNA levels were significantly different between R2 and A/J mice, and only *Nrcam* mRNA levels were significantly different between R3 and A/J mice. These results suggest that *Zfp125* and *Nrcam* in epididymal fat are candidate genes in *Fl1sa*.

## Discussion

We previously found that SMXA-5 mice developed a fatty liver induced by a high-fat diet, and further identified *Fl1sa* in the region centromere-53.06 Mb on chromosome 12 as the fatty liver QTL. We then determined the A/J-derived allele of *Fl1sa* to be the cause of fatty liver. In this study, we chose a congenic mapping strategy to narrow the *Fl1sa* region and conducted a microarray analysis to identify candidate genes in *Fl1sa*.

First, we narrowed the chromosomal region of *Fl1sa* because the chromosomal region spanned by *Fl1sa* was extremely broad (centromere-53.06 Mb, 611 genes). Therefore, we established the R2 and R3 congenic strains (Fig. 1) and then analyzed the liver lipid accumulation of these congenic mice that were fed the high-fat diet for 7 weeks. Final body weight in A/J-12^SM^ and R3 mice was significantly lower than that in A/J mice, even though food intake was the same between the R3 congenic mice and the A/J mice (Table 1). Liver triglyceride content in A/J-12^SM^, R2, and R3 mice was significantly lower than that in A/J mice (Fig. 2a); however, the liver total lipid content in R2 and R3 mice was not significantly changed compared to that in A/J mice (Fig. 2b). The results of liver lipid analysis showed that R2 and R3 mice had a smaller effect on fatty liver compared to A/J-12^SM^ mice. Both serum triglyceride and total cholesterol concentrations were not different in R2 and R3 mice from those in A/J mice (Fig. 2c and d). In both congenic mice, serum lipid levels did not correlate with the accumulation of triglycerides in the liver. These results suggest that at least one of the genes responsible for fatty liver exists within the chromosomal region of the SM/J allele in R2 (centromere-29.20 Mb) and R3 (29.20–46.75 Mb) mice, respectively. The genes in the R2 and R3 regions were not found to be effective in altering the serum lipid concentration; however, genes in the R3 region might affect body weight gain. Moreover, genes in these regions might interact with each other because liver triglyceride and liver total lipid content in R2 and R3 mice were not as low as those in A/J-12^SM^ mice.

Second, we attempted to identify candidate genes in *Fl1sa* in the R2 and R3 chromosomal region. We previously used DNA microarray to analyze the comprehensive gene expression of livers in A/J and A/J-12^SM^ mice that were fed the high-fat diet for 7 weeks [9]. In this study, we refined the list of genes in this region whose expression levels in the liver were different between A/J and A/J-12^SM^ mice (<0.60-fold or >1.68-fold) in the R2 and R3 regions (centromere-29.20 Mb and 29.20–46.75 Mb, respectively). We explored candidate genes having cis-acting expression in each chromosomal region of R2 and R3 mice. Consequently, we succeeded in isolating two genes, *Iah1* (21.31 Mb) and *Rrm2* (24.70 Mb), as candidate genes in the R2 region (Fig. 5a). *Iah1* was identified as an esterase that exhibits hydrolytic activity against acetate esters in yeast [12]. We previously reported that mouse *Iah1* mRNA is broadly expressed in the liver, kidney, epididymal fat, lung, spleen, and muscle [9]. Furthermore, stable overexpression of mouse Iah1 in Hepa1-6 cells suppressed the mRNA expression of lipid metabolism-related genes *Cd36* and *Dgat2* [9]. Our data suggested that mouse Iah1 prevents the development of fatty liver by suppressing *Cd36* and *Dgat2* gene expression. Thus, we suggest that high expression of *Iah1* contributes to the decreased liver triglyceride content in R2 congenic mice. *Rrm2* encodes the β-subunit of the protein ribonucleotide reductase, a rate-limiting enzyme involved in *de novo* dNTP biosynthesis [13]. It was reported that RRM2 overexpression was observed in various types of cancer [13], and that patients with liver cirrhosis and hepatocellular carcinoma exhibited high levels of RRM2 [14]. However, the relationship between *Rrm2* and fatty liver has not been clarified. From the present results, we identified *Iah1* and *Rrm2* as candidate genes in the liver for the development of fatty liver.

On the other hand, liver weight and liver triglyceride content are inversely correlated with epididymal fat weight (Fig. 3a and b). These results suggest that controlling the epididymal fat weight contributes to triglyceride accumulation in the liver. In addition, it was reported that liver lipid accumulation and epididymal fat weight showed inverse correlation in other mouse strains [15, 16]. To identify differentially expressed genes in epididymal fat, we performed DNA microarray analysis using RNAs from epididymal fat of A/J and A/J-12^SM^ mice. We detected 19 genes, whose expression levels in epididymal fat were changed between A/J and A/J-12^SM^ mice (<0.60-fold or >1.68-fold) within the R2 and R3 chromosomal regions (Table 2). Subsequently, we validated 6 candidate genes whose mRNA levels in A/J-12^SM^ mice were significantly different from those in A/J mice: *Ntsr2* (16.65 Mb), *Zfp125* (20.89 Mb), *Gdap10* (32.82 Mb), *Nrcam* (44.32 Mb), *Stxbp6* (44.85 Mb), and *Nova1* (46.69 Mb) (Fig. 4a and b). Finally, we selected *Zfp125* (20.89 Mb, in R2 region) and *Nrcam* (44.32 Mb, in R3 region) as candidate genes in epididymal fat, because their mRNA levels in R2 and R3 congenic mice were significantly different in accordance with the change in A/J-12^SM^ mice compared to A/J mice (Fig. 5b and c). *Zfp125* is a zinc finger protein expressed in many tissues and might be involved in the regulation of cellular processes such as cell proliferation and transformation [17]. *Nrcam* is a neuronal cell adhesion molecule that mediates neuron-neuron and neuron-glia adhesion [18]. Furthermore, it was reported that *Nrcam* was induced in the liver of Fisher-344 rats fed 2-aminoanthracene [19]. Further investigation is needed to clarify the functions of these two genes in lipid metabolism.

The R2 and R3 regions on mouse chromosome 12 are syntenically conserved in three regions on human chromosomes 2, 7, and 14. In these syntenic regions, human Genome Wide Association Studies (GWAS) for metabolic syndrome identified 205 SNPs, which were shown in GWAS catalog (NHGRI-EBI Catalog of published genome-wide association studies, http://www.ebi.ac.uk/gwas/home). However, there are no SNPs associated with metabolic syndrome in 4 candidate genes (*Iah1*, *Rrm2*, *Zfp125*, and *Nrcam*).

Moreover, we previously performed exome analysis in A/J and SM/J (data were deposited in DDBJ Sequence Read Archive, Accession No. DRA002145). We identified non-synonymous SNPs between A/J and SM/J mice (48 genes) in the genomic region R2 and R3 from the exome data (Additional files 4 and 5). The genes having non-synonymous SNPs might cause the change in the function of their translated proteins. We also consider these genes as potential candidate genes for *fl1sa*. At present, we cannot assess the importance of amino acid substitutions in each gene. In *Iah1* gene, we confirmed the two non-synonymous SNPs and these SNPs lead to amino acid substitutions (S37A and G75E in A/J strain, Additional files 4 and 5). Other candidate genes identified by our DNA microarray analysis did not have the non-synonymous SNPs.

## Conclusions

In this study, by using R2 and R3 congenic mice, we identified *Iah1* and *Rrm2* from the liver, and *Zfp125* and *Nrcam* from epididymal fat as candidate genes in *Fl1sa*. Although the relationship between these genes and fatty liver has not been previously reported, we demonstrated that *Iah1* overexpression affected the expression of lipid metabolism-related genes in Hepa1-6 cells [9]. Therefore, at present, we are examining the incidence of fatty liver in *Iah1*-knockout mice. We hypothesize that ectopic fat accumulation in the liver was brought by the organ-organ interaction between the liver and epididymal fat. Thus, *Zfp125* and *Nrcam* in epididymal fat might affect liver triglyceride accumulation through the regulation of epididymal fat weight. In future experiments, to uncover the molecular mechanism underlying the relation between these candidate genes and lipid metabolism, we will need to perform overexpression or knockdown experiments using candidate genes in hepatocytes or adipocytes. Overall, this study is the first step to elucidating the mechanism of fatty liver development coinciding with changes in fat distribution.

## Additional files

Additional file 1: Sequences of primers used for genotyping. (DOCX 14 kb)
Additional file 2: Expression profiles of all genes in R2 and R3 regions. (XLSX 43 kb)
Additional file 3: Sequences of primers used for real-time RT-PCR. (DOCX 21 kb)
Additional file 4: Allelle variants on chromosome 12 (Centromere-46.75 Mb) in A/J strain versus C57BL/6 strain. (XLSX 15 kb)
Additional file 5: Allelle variants on chromosome 12 (Centromere-46.75 Mb) in SM/J strain versus C57BL/6 strain. (XLSX 13 kb)

## Abbreviations

APOC3: Apolipoprotein C-3
Cd36: Cd36 antigen
Dgat2: Diacylglycerol O-acyltransferase 2
*Fl1sa*: Fatty liver 1 in SMXA RI strains
Gdap10: Ganglioside-induced differentiation-associated-protein 10
Iah1: Isoamyl acetate-hydrolyzing esterase 1 homolog (*Saccharomyces cerevisiae*)
NAFLD: Nonalcoholic fatty liver disease
Nova1: Neuro-oncological ventral antigen 1
Nrcam: Neuronal cell adhesion molecule
Ntsr2: Neurotensin receptor 2
PLIN1: Perilipin-1
PNPLA3: Patatin-like phospholipase domain containing protein 3
Prkd1: Protein kinase D1
QTL: Quantitative trait locus
RI: Recombinant inbred
Rrm2: Ribonucleotide reductase M2
SOX4: SRY (sex determining region Y)-box 4
Stxbp6: Syntaxin binding protein 6 (amisyn)
Zfp125: Zinc finger protein 125
