# Supplementary material for: Genetic dissection of the fatty liver QTL Fl1sa by using congenic mice and identification of candidate genes in the liver and epididymal fat
Source: BMC Genet. 2016 Nov 17;17:145. doi: 10.1186/s12863-016-0453-7 (PMC5114839; doi:10.1186/s12863-016-0453-7)
Supplement: Additional file 1: — Sequences of primers used for genotyping. (DOCX 14 kb) [file 12863_2016_453_MOESM1_ESM.docx]

Additional file 1

Sequences of primers used for genotyping

| Sequence name | Primer sequence | |
| --- | --- | --- |
| D12Mit84 | Forward:  Reverse: | ATA AGT TAG GGG AAA TCA CTG  GGT GTG GCT TTC CCA AAC TA |
| D12Mit85 | Forward:  Reverse: | GTA CCA AGG GGT CAT GAG GA  AAT GGG GCT GAA ACA ATA CG |
| D12Mit270 | Forward:  Reverse: | AGG CAT CTT TTT GAA TAG TTT TAT ACA  ATT AAG GCA TTG GTA AAG TGA TAT ATG |
| rs29184291 | Forward:  Reverse: | TGG GTC TAT GAG GGT CGT TT  GGC CAA CAT CCG TAC CAT AC |
| D12Mit112 | Forward:  Reverse: | CTT CAG GCC TCC CTG GTA C  TGC CTC CAA ATA TAC TCA CAA GC |
| D12Mit110 | Forward:  Reverse: | CGA CTC CCG AAA CAC TCT TC  TGC AGT GGG CAT ACT TTC TG |
| D12Mit36 | Forward:  Reverse: | CAT CAC ACC AGG TTT AGA ATT TT  AGG CAC TCT TCT GAC CTC CA |
| Akap6  (rs29155520) | Forward:  Reverse: | CTA TGC CGA CGT GTG TGG TA  AAA AGC AAT GTT TTC CCC ATA A |
