# Supplementary material for: Genetic dissection of the fatty liver QTL Fl1sa by using congenic mice and identification of candidate genes in the liver and epididymal fat
Source: BMC Genet. 2016 Nov 17;17:145. doi: 10.1186/s12863-016-0453-7 (PMC5114839; doi:10.1186/s12863-016-0453-7)
Supplement: Additional file 3: — Sequences of primers used for real-time RT-PCR. (DOCX 21 kb) [file 12863_2016_453_MOESM3_ESM.docx]

Additional file 3

Sequences of primers used for Real-time RT-PCR.

| Symbol | Primer sequence | | Accession ID |
| --- | --- | --- | --- |
| *Actb*  (*β-actin*) | Forward:  Reverse: | AGA TGA CCC AGA TCA TGT TTG AGA  CAC AGC CTG GAT GGC TAC GT | NM_007393.3 |
| *Rrm2* | Forward:  Reverse: | CGC CGA GCT GGA AAG TAA AG  TTT CTC TCA GTA ACG GCT CAT CCT | NM_009693 |
| *Pfn4* | Forward:  Reverse: | CGC GAA GGG AAG GGT TGT  AGT CAT CTG CCC GAA CAC ATT | NM_028376 |
| *Fkbp1b* | Forward:  Reverse: | TGG AGG GAC TTG AGC CAG TT  AAA GTG AGC AGC CAA CAG AAG AT | NM_016863 |
| *Apob* | Forward:  Reverse: | TGG GAT TCC ATC TGC CAT CTC GAG  GTA GAG ATC CAT CAC AGG ACA ATG | NM_009693 |
| *Nt5c1b* | Forward:  Reverse: | CGC CCA AAC CCA AGC AT  CAT ATT GAA AAG TGC CCG AGA AG | NM_027588 |
| *Ntsr2* | Forward:  Reverse: | ACC CAG CGC AGT CAT TGA C  AGT TGA GGG CAG GAG GTG TTA C | NM_008747 |
| *Zfp125* | Forward:  Reverse: | GCG TCC AGT AGC AGG GTT ACA C  CTG GAC CGT GCG CTT TG | AJ005350 |
| *Rsad2* | Forward:  Reverse: | TGG CCT GGA ACT CAT GAT ACC  ACG TGC AAT CCC TAG CAC TTG | NM_021384 |
| *Cmpk2* | Forward:  Reverse: | GCT TAA CTC TGC GGT GTT CCA  AAA GGA TGT GCA CTC TTC CAA GA | NM_020557 |
| *Allc* | Forward:  Reverse: | GGA CAG ACC CCC GGT GTT A  AAC CCG GGA CCA GGA GAA | NM_053156 |
| *Sh3yl1* | Forward:  Reverse: | GCG TCT TCC GGA TGG AAA  CAA GCC CAG CTA TCC CAA TG | NM_013709 |
| *Slc26a3* | Forward:  Reverse: | CCG TGG TTG GGA ACA TGA GT  AAA CTT CCA CGC TGG GTG TAA | NM_021353 |
| *Gdap10* | Forward:  Reverse: | TGC ACC CAC AGT GAA CAC ATT  GGA AGC GTA ACC TTG TTG GAG TA | BC052902 |
| *Cdhr3* | Forward:  Reverse: | TCT GCA AAT GGC ACC CTC TT  AGC TCT TGT GCC CTG CTT CA | NM_001024478 |
| *Efcab10* | Forward:  Reverse: | CGG CAG AGG CTG CAT ATC AT  TGC AGT ACA CAG ACC CAA GTT TTT | NM_029152 |
| *Dgkb* | Forward:  Reverse: | GTT TGC AGA TCA CTC CTG TTC CT  CCC ACC ACT TTT AGG GTT GAC A | NM_178681 |
| *Nrcam* | Forward:  Reverse: | GCA CTA AAG GAA GCG CTC TTC A  TTG GGC CAC GGG AAT TT | NM_176930 |
| *Stxbp6* | Forward:  Reverse: | CCC AGC AGT TTG CAG AAA CC  GGA TAG GCA GTT TCT CAA CAT TTA TG | NM_144552 |
| *Nova1* | Forward:  Reverse: | GCC ATC TTC CCC AAC TAC CA  CTG TGC TGT TGG GAA CTA TAA TCT TT | NM_021361 |
